# Anti-PD1 Consolidation in Patients with Hodgkin Lymphoma at High Risk of Relapse after Autologous Stem Cell Transplantation: A Multicenter Real-Life Study

**DOI:** 10.3390/cancers14235846

**Published:** 2022-11-27

**Authors:** Rosaria De Filippi, Gianpaolo Marcacci, Enrico Derenzini, Maurizio Musso, Daniela Donnarumma, Emanuela Morelli, Caterina Patti, Alessio Maria Edoardo Maraglino, Renato Scalone, Luigia Simeone, Cristina Becchimanzi, Sara Mele, Stefania Crisci, Fortunato Morabito, Antonio Pinto

**Affiliations:** 1 Department of Clinical Medicine and Surgery, Università degli Studi Federico II, 80131 Naples, Italy; 2 Hematology-Oncology and Stem Cell Transplantation Unit, Istituto Nazionale Tumori, Fondazione ‘G. Pascale’, IRCCS, 80131 Naples, Italy; 3 Oncohematology Division, IEO European Institute of Oncology IRCCS, Department of Health Sciences, University of Milan, 20129 Milan, Italy; 4 Department of Oncology, Hematology and BMT Unit, Casa di Cura La Maddalena, 90145 Palermo, Italy; 5 Division of Onco-Hematology, Azienda Villa Sofia-Cervello, 90146 Palermo, Italy

**Keywords:** autologous stem cell transplantation, consolidation, PD1-blockade, refractory/relapsed Hodgkin lymphoma

## Abstract

**Simple Summary:**

Autologous stem cell transplantation (ASCT) represents a standard strategy for patients with relapsed and refractory (RR) Hodgkin Lymphoma (HL). Unfortunately, patients displaying some pre-transplant adverse predictors eventually progress, requiring further treatments and several succumb to their lymphoma. This retrospective multicenter study aimed to evaluate the effectiveness of programmed death receptor-1 (PD-1)-blockade as a consolidation treatment for patients with RR-HL at high risk of ASCT failure. We collected data from 26 patients with ≥2 risk factors for recurrence who had received anti-PD1 consolidation after ASCT. Patients received a median of 13 consolidation courses (range 6–30), without toxicity-related discontinuations. At a median follow-up of 25.8 months post-ASCT, the median progression-free (PFS) was 42.6 months, with a 2-year PFS and overall survival rates of 79% and 87%, respectively. Post-ASCT consolidation with anti-PD1 is feasible and effective and may represent a valuable management option for patients at high risk of recurrence.

**Abstract:**

(1) Background: Consolidation therapy is an emerging strategy for patients with relapsed/refractory (RR) Hodgkin Lymphoma (HL) at high risk of failing salvage autologous stem cell transplantation (ASCT). (2) Objectives: To assess the safety and effectiveness of PD1-blockade consolidation for these high-risk patients. (3) Design: Multi-center retrospective analysis. (4) Methods: We identified 26 patients given anti-PD1 consolidation, from June 2016 to May 2020. (5) Results: Patients displayed the following risk factors: refractory disease (69%), relapse < 12 months from upfront therapy (15%), ≥2 lines of salvage therapy (73%), extranodal disease (65%). Nineteen patients (73%) had ≥3 of these factors. In addition, 16 patients (61%) also displayed PET-positive (Deauville ≥ 4) disease before ASCT. Treatment-related adverse events (TRAEs), never graded > 3, occurred in 12 patients (46.15%) and mainly included skin rashes (41.7%), transaminitis (33.3%), and thyroid hypofunction (25%). Patients completed a median of 13 courses (range 6–30). At a median follow-up of 25.8 months post-ASCT, the median progression-free (PFS) was 42.6 months, with a 2-year PFS and overall survival rates of 79% and 87%, respectively. (6) Conclusions: Post-ASCT consolidation with anti-PD1 is feasible and effective. Further studies are warranted to define the optimal treatment length and patients’ subsets more likely to benefit from this approach.

## 1. Introduction

High-dose chemotherapy (HDT) followed by autologous stem cell transplantation (ASCT) is the standard of care for patients with relapsed and refractory (RR) classic Hodgkin Lymphoma (cHL) [1,2]. Unfortunately, disease recurrence remains the major cause of ASCT failure. Several clinical risk factors, encompassing response patterns to frontline therapy, the efficacy of pre-ASCT salvages, and disease status at transplant, have been historically defined as negative predictors of post-ASCT outcomes [3,4,5]. Namely, primary disease refractoriness, early relapse (<12 months from upfront therapy), extranodal disease and/or B symptoms at relapse, need for ≥2 pre-transplant salvage therapies, and persistence of fluorodeoxyglucose (FDG)-avid disease, by positron emission tomography (PET), before HDT are statistically associated with poor post-ASCT survival [6,7,8,9]. Therefore, patients displaying 2 or more of these factors represent a population at a high risk of ASCT failure [9,10].

Recently, the historically grim outlook of these patients has been markedly improved by the introduction of CD30-targeted agents, such as brentuximab vedotin (BV), and programmed cell death-1 (PD-1)-blockers, including Nivolumab and Pembrolizumab [11,12,13,14]. In particular, the unique biology of HL renders this tumor the most responsive malignancy to PD-1 blockade. This fact is mainly due to recurrent alterations of the 9p24.1 chromosomal region, which harbors PD-L1 and PD-L2 loci, carried by tumor cells in almost all HL cases [15]. Even though the exact biologic mechanism through which anti-PD1 antibodies reshape the HL microenvironment and restore anti-tumor immune response is still incompletely understood, the high density of PD-L1/-L2 expression on neoplastic cells of HL may play a dominant role [15,16].

Patients given BV or PD1-blockers after ASCT failure can achieve progression-free survival (PFS) rates approaching 2 years and 5-year overall survival (OS) rates up to 70–80% [11,14,15,16,17,18]. Then, different approaches were pursued to employ BV and PD-1 blockers to prevent ASCT failure, including early use in patients with a PET-positive status after salvage chemotherapy, application as single agents, followed by chemotherapy in case of an inadequate response pre-ASCT, direct integration into salvage regimens and combined use of BV and anti-PD1 antibodies as a ‘no chemotherapy salvage’ [12,19,20,21,22,23]. A further strategy is based on early intervention with post-ASCT consolidation to prevent disease recurrence. A 5-year follow-up analysis of the AETHERA randomized study showed that post-ASCT consolidation with BV yields a 48% reduction in the risk of disease progression over placebo and that such benefit is more pronounced in patients with ≥2 pre-transplant risk factors [9,24].

Consolidation with PD-1 blockade is another approach to achieve improved disease free survival in these high risk patients. Armand and coworkers showed that post-ASCT consolidation with single-agent pembrolizumab in patients with high RR-cHL yields an 18-month PFS of 82% with an OS of 100%. No unexpected safety signals nor disturbances in post-transplant immune reconstitution were detected [25,26]. Based on these results, other PD-1 blockers, such as nivolumab, alone and in combination with BV, are currently being tested as post-ASCT consolidation in patients with high-risk RR-HL [27,28].

To the best of our knowledge, the safety and effectiveness of PD-1 blockade post-ASCT were not yet assessed in a real-world setting. To this end, we conducted a retrospective multicenter study of high-risk patients with RR-HL who had received PD-1 blockers as consolidation after ASCT.

## 2. Methods

### 2.1. Patients and Study Procedures

We retrospectively identified 26 patients with RR-HL at high risk of ASCT failure who received nivolumab as post-ASCT consolidation treatment at 4 transplant Centres in Italy from June 2016 to May 2020. The study was conducted according to the Declaration of Helsinki and data collection and analysis was performed under the institutional ethical and review board-approved protocol LYMRO-22 (20/22 OSS; date of approval 27 July 2022, Istituto Pascale, Italy) and the INTHEMA study protocol (v 2.0 IRSTB100, NCT04298892, date of approval 27 January 2022). We identified patients using clinical databases and pharmacy treatment plan records at the different Centres (Supplementary Figure S1). Anti-PD1 consolidation was proposed based on clinical risk factors for ASCT failure and treatment was delivered as an off-label therapy after written informed consent. For this study, high risk for post-ASCT relapse was defined by ≥2 of the following criteria: primary refractory disease (progression during upfront chemotherapy, or only transient response, either complete or partial lasting ≤ 90 days), relapse within 12 months from completing frontline chemotherapy, extranodal disease and/or B symptoms at relapse, administration of >2 pre-transplant salvage chemotherapy lines, presence of FDG-avid disease, i.e., 5-points Deauville score (DS) > 3, at last PET scan before transplant. Records of patients not meeting the above criteria were excluded from this analysis, as well as those patients who, despite fulfilling the risk factors criteria, had clinical and/or radiographic evidence of disease progression after ASCT but before starting anti-PD-1 consolidation. Prior exposure to BV or PD-1 blockers (nivolumab or pembrolizumab) before HDT was allowed. Criteria for ASCT eligibility, mobilization procedures, leukapheresis modalities, choice, and administration of conditioning HDT, were based on standard guidelines of participating institutions. Similarly, the length of post-ASCT PD1-blockade and reasons for its discontinuation, frequency of imaging assessments, and type of further therapy, if any, was at the discretion of physicians from participating centers. All patients’ information has been de-identified for this study.

### 2.2. Efficacy and Toxicity Assessments

The primary study endpoint was PFS, which was calculated from the date of stem cell reinfusion to the time of relapse, disease progression, or death, whichever occurred first. The secondary endpoints were OS, calculated from the date of ASCT to the last follow-up visit at database lock, response rates, and occurrence of Treatment-related adverse events (TRAEs), graded according to the Common Toxicity Criteria (CTCAE; v. 5.0). Complete response (CR), partial response (PR), stable disease (SD), and progressive disease (PD) were defined according to Lugano criteria, based on the first available PET scan after ASCT [29]. Imaging with FDG-PET was considered negative if DS ≤ 3 and positive if DS ≥ 4.

### 2.3. Statistical Methods and Analyses

Statistical analyses were conducted as previously described in detail [30]. Briefly, data are expressed as absolute numbers and percentages. Statistical comparisons were performed using two-way tables for the Fisher’s exact test and multi-way tables for Pearson’s Chi-square test for categorical variables. Mann–Whitney U test was utilized for the comparison between two groups of cases on the same variable. PFS and OS analyses were performed using the Kaplan–Meier method. Statistical significance of associations between individual variables, including pre-ASCT risk factors, and PFS or OS was calculated using the log-rank test. Univariate Cox regression analyses investigated the prognostic impact of the outcome variables. In the Cox models, data were expressed as hazard ratios (HR) and 95% confidence intervals (CI) and displayed as a Forest plot. A value of *p* < 0.05 was considered significant. All analyses were performed by SPSS for Windows Version 22, Chicago, Illinois, USA & STATA 13 for Windows StataCorp (Lakeway Drive, College Station, TX, USA).

## 3. Results

### 3.1. Patient Characteristics

The clinical characteristics of the 26 patients included in the study are summarized in Table 1. The median age at ASCT was 30 years (range, 19–58 years), and 15 (58%) were males. Frontline treatment was ABVD in the vast majority of cases (96%) and 10 patients (38%) had received upfront consolidation radiotherapy. Five patients (19%) had also failed interim PET-driven chemotherapy escalation to BEACOPP, after 2 courses of ABVD.

### 3.2. Risk factors for ASCT Failure

As detailed in Table 1, our patients had received a median number of 4 (range, 1–5) systemic treatments before transplant and the vast majority of them (73%) displayed ≥3 risk factors for post-ASCT recurrence.

### 3.3. Pre-ASCT Exposure to BV and PD-1 Blockers

All patients but one received a median number of 4 BV courses (range: 2–8), while 61% of patients were previously exposed to PD-1 blockers for a median number of 11 courses (range: 4–32). More than half of the patients had received both these agents before ASCT (Table 1).

### 3.4. Disease Status at Transplant

Overall, 16 patients (61%) had a PET-positive status (DS ≥ 4) before HDT, with 14 cases in PR (54%) and 2 (8%) in SD/PD according to Lugano criteria. Ten patients (38%) were conditioned in complete metabolic response (DS ≤ 3). Nine of these patients had received anti-PD1 as the last salvage line before ASCT. Conversely, 50% of patients in PR at transplant were given anti-PD1 as the last treatment line before conditioning. Two patients (1 in SD and 1 in PD), never received prior anti-PD1 (Table 1).

### 3.5. Conditioning Regimens

The great majority of patients (n = 23; 88%) received the FEAM (fotemustine, etoposide, cytarabine, melphalan) regimen; two were conditioned with BEAM (carmustine, etoposide, cytarabine, melphalan) and one with busulphan plus fludarabine.

### 3.6. Post-ASCT PD1-Blockade

Patients initiated consolidation at a median of 44 days (range, 10–129 days) from ASCT and received a median of 13 courses (range, 6–30) of nivolumab (240 mg IV flat dose, every 2 weeks). In 18 cases (69%) nivolumab was discontinued upon physicians’ decision due to sustained CR and patients underwent follow-up after having received a median of 15 courses of nivolumab (range, 6–30). The median time to discontinuation for these patients was 7.6 months (range, 2.3–15.5). None of the patients who discontinued nivolumab while in CR progressed at the last follow-up, leading to a not reached median duration of overall response (CR + PR). However, 76.7% and 64.5% of patients were still in response at 1 and 2 years, respectively. Five patients (19%) discontinued consolidation due to disease progression or stability, one (4%) was bridged to allotransplantation and two patients were in uninterrupted nivolumab at their last follow-up (4.2 months and 6.9 months from ASCT and 2.8 and 5.6 from treatment initiation). No treatment discontinuations due to toxicity were recorded.

### 3.7. Survival Outcomes and Predictors

**Figure 1:**
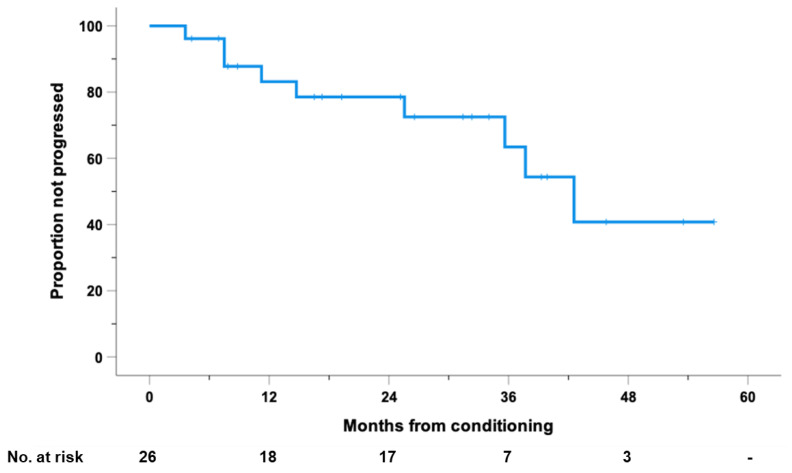
Progression-free survival (PFS), calculated from the day of conditioning, for the entire cohort of 26 high-risk patients with RR-HL who received PD1-blockade after autologous stem cell transplantation (ASCT).

At 25.8 months of median follow-up (range, 4.2–63.1) after ASCT, 9 patients progressed or died for any cause; the estimated median PFS was 42.6 months, with 1- and 2-year PFS rates of 83% (95% CI: 68 to 98) and 79% (95% CI: 62 to 95), respectively (Figure 1).

**Figure 2:**
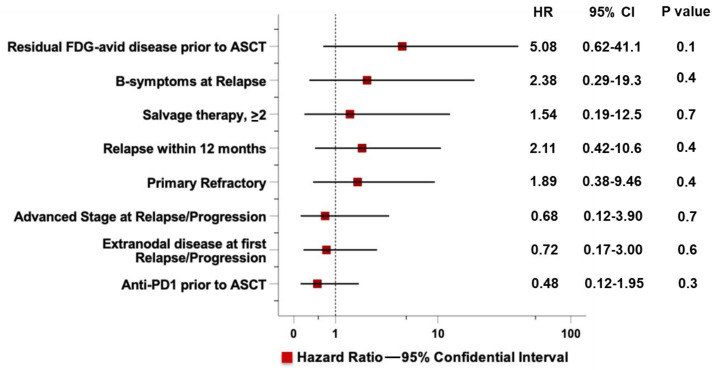
Cox univariate analyses of progression-free survival for 26 high-risk patients with RR-HL who received PD1-blockade after autologous stem cell transplantation (ASCT). The Forest plot summarizes the Cox univariate analysis for risk factors associated with post-ASCT progression.

A univariable analysis to evaluate the prognostic impact of main risk factors including primary disease status (chemorefractoriness or early relapse), extranodal sites and B-symptoms at relapse, administration of ≥ 2 salvage therapy lines, and PET-positive status (DS ≥ 4) before HDT and pre-ASCT exposure to anti-PD1, failed to identify a statistically significant predictor for PFS (Figure 2).

**Figure 3:**
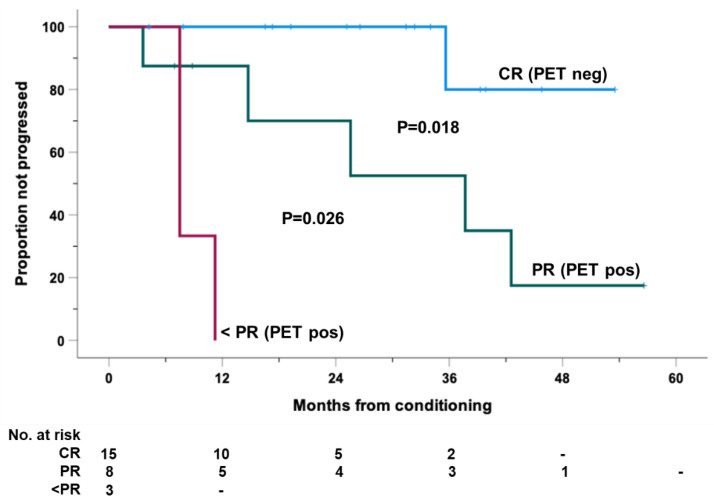
Progression-free survival (PFS), calculated from the day of conditioning, for the entire cohort of 26 high-risk patients with RR-HL who received consolidation with PD1-blockade after autologous stem cell transplantation (ASCT). Cases were clustered according to the achievement of CR (PET neg; DS ≤ 3), PR and less than PR (PET pos; DS ≥ 4) at the first imaging evaluation after ASCT and before start of PD1-blockade.

In contrast, landmark analyses evidenced that the attainment of the CR status (PET negative; DS ≤ 3) at the first post-transplant disease assessment before starting of PD1-blockade [at a median of 1.65 months from ASCT (range, 0.10-3.40)] was a statistically significant predictor of a longer PFS outcome. All of these patients were progression-free at 1- and 2-year, while those in PR (PET positive; DS ≥ 4) had 1- and 2-year PFS rates of 87% (95% CI: 65 to 100) and 70% (95% CI: 34 to100), respectively (Figure 3). The only three patients with less than PR (2 SD, 1 PD) had the worst PFS rates.

**Figure 4:**
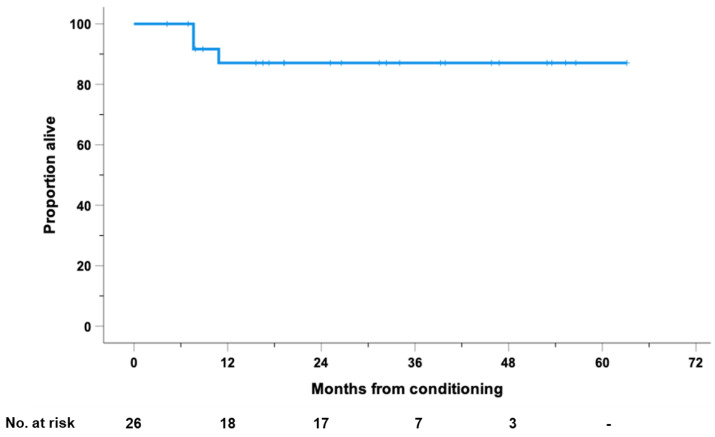
Overall survival (OS), calculated from the day of stem cell reinfusion, for the entire cohort of 26 high-risk patients with RR-HL who received consolidation with PD1-blockade after autologous stem cell transplantation (ASCT).

The median OS after ASCT was not reached for the entire cohort, with 1- and 2-year OS rates of 87% (95% CI: 73 to 100) (Figure 4).

**Figure 5:**
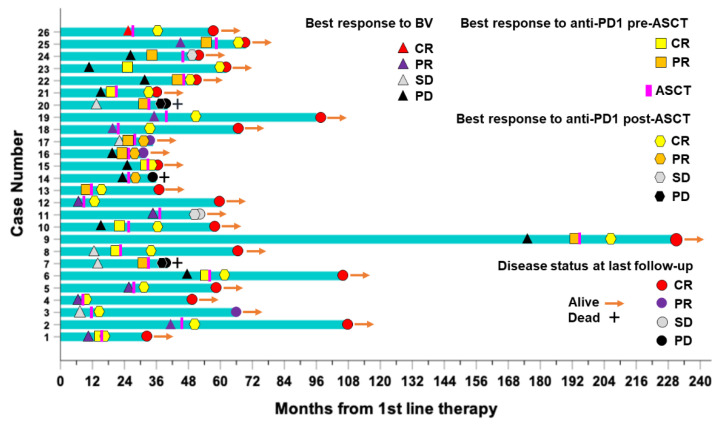
Swimmer’s plot of 26 high-risk patients with RR-HL who received consolidation with PD1-blockade after autologous stem cell transplantation (ASCT). Best responses (CR, complete response; PR, partial response; SD, stable disease; PD, progressive disease) to brentuximab vedotin (BV) and anti-PD1 treatment before (pre-ASCT) and after ASCT (post-ASCT), disease status and survival (alive/dead) at last follow-up are indicated accordingly. Bars represent the timeline (months) from upfront therapy for each patient included in the study.

A swimmer plot recapitulating the clinical timelines for all 26 patients included in the study is shown in Figure 5.

### 3.8. Safety Analysis

**Figure 6:**
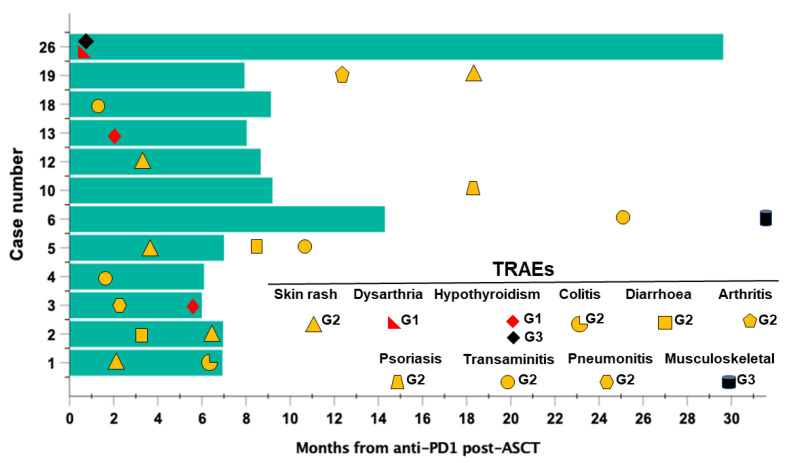
Timelines of occurrence and grading of treatment-related adverse events (TRAEs) registered in patients (n = 12) with high-risk RR-HL during post-ASCT consolidation with PD1-blockade. Bars represent the time (months) of exposure to the anti-PD1 antibody nivolumab. Symbols represent TRAEs grade according to the Common Toxicity Criteria (CTCAE; v. 5.0).

During post-ASCT PD1-blockade, a total of 12 patients (46.15%) developed one or more TRAE of grade G1-G2 and never higher than G3 (Figure 6).

The most frequent events included skin rashes (n = 5; 41.7%), transaminitis (n = 4; 33.3%) and thyroid hypofunction (n = 3; 25%). Events were managed according to ESMO guidelines and none led to momentary or permanent treatment discontinuation [30,31]. Seven patients displayed more than one TRAE. Interestingly, in 4 patients, events occurred after treatment was discontinued for toxicity-unrelated reasons at a median of 28.6 months (range, 18.6–33.3) from the last anti-PD1 dose and after a median of 13 anti-PD1 cycles (range, 10–16 cycles) (Figure 6). In all of these patients (namely #19, #10, #6, #5) PD1-blockade was discontinued upon physician decision due to a sustained CR.

## 4. Discussion

To our knowledge, this is the first ‘real-life’ study assessing the safety and effectiveness of PD-1 blockade as a consolidation strategy in patients with RR-cHL at high risk of ASCT failure. Our study population included 73% of patients with ≥3 established risk factors for post-ASCT recurrence and 61% had PET-positive (Deauville ≥ 4) disease before transplant. Treatment was well tolerated, without discontinuations due to toxicity, and we documented a median post-transplant PFS of 42.6 months, with 2-year PFS and OS rates of 79% and 87%, respectively, for the whole cohort.

Consolidation started at a median of 44 days from the conditioning regimen, even though, as expected for a real-life study, we had 3 outlier patients with a longer time interval. These specific cases had a confirmed PET-negative status early before the start of consolidation, excluding an intercurrent disease progression. One of them recurred after 10 consolidation doses. We were then unable to estimate the relative contribution of HDT per se to the survival outcomes of the remaining 2 patients.

The potential of PD1-blockade to prevent disease recurrence after ASCT in high-risk patients with RR-HL was first documented by Armand et al. [25]. These investigators reported an 18-month PFS of 82% with an OS of 100% in the 28 evaluable patients from a phase 2 study. Twenty-seven (90%) and 12 (40%) of these patients, had at least 1 or 2 of the AETHERA study-defined risk factors for ASCT failure [9,25]. No unexpected safety signals were detected nor significant disturbances in post-transplant immune reconstitution kinetics [25]. Instead, consolidation with pembrolizumab was associated with earlier recovery of natural killer (NK) cells and a sustained elevation of circulating plasmacytoid and immature dendritic cells, whose possible impact on disease control remains to be determined [25,26].

We are unable to compare the results of our real-life analysis with the pivotal phase 2 study of Armand et al. due to differences in study design, inclusion criteria, risk profiles and duration of anti-PD1 exposure [25]. While this trial only accrued patients with chemosensitive disease at transplant, i.e., CR or PR, 60% of our patients had active disease at ASCT [25]. An additional relevant difference is that a number of our patients (42%), had metabolically active disease sites after the transplant and before starting the PD1-blockade. By separately analyzing these cases it emerged that those patients who received anti-PD1 while in CR (PET negative disease) displayed the best PFS outcomes as compared with those in PR or less. These findings further support that a true anti-PD1 consolidation strategy should be optimally applied to patients with metabolically silent disease after ASCT [25]. The rate of pre-ASCT exposure to PD-1 blockade was also different in our series. While only 20% of patients were exposed to anti-PD1 agents in the study of Armand et al., 58% of our patients received nivolumab or pembrolizumab before ASCT. In these cases, anti-PD1 was the last salvage therapy before transplant [25].

While our patient population had a significant load of risk factors and prior treatments, together with a substantial rate of uncontrolled (PET-positive) disease at transplantation, the high rate of pre-ASCT exposure to anti-PD1 might have biologically counterbalanced these adverse factors by promoting a better efficacy of HDT conditioning. Several studies, in patients with chemorefractory RR-HL, have documented that treatment with anti-PD1, likely due to a clonal ‘reshaping’ effect, enhance/restores sensitivity to anticancer agents, including those present in the conditioning regimens for ASCT [32,33,34]. In addition, an important real-life study indicated that using PD-1 blockade as a bridge to ASCT in chemorefractory patients was associated with excellent post-transplantation outcomes, without any apparent improvement by post-ASCT treatment with anti-PD1 [35]. Therefore, we cannot exclude that the favorable post-ASCT outcomes registered in our patients, can be also partly due to the enhanced efficacy of pre-transplant HDT prompted by previous anti-PD1 exposure [35]. It is to note, however, that 8 out of 10 patients who never received pre-transplant PD1-blockade, achieved comparable outcomes, in terms of post-ASCT response and survival, with those who did. On the other hand, the occurrence of delayed TRAEs in some of our patients, long after treatment withdrawal, further testifies the prolonged pharmacodynamic effects of PD1-blockers, as highlighted by previous studies [36,37]. This concept may further support PD1-blockade as a valuable strategy for post-ASCT consolidation.

Another important issue is the optimal duration of consolidation. Our retrospective analysis revealed that most patients (69%) discontinued treatment based on the physician’s decision, due to a sustained post-transplant CR. None of these patients, who had received a median of 15 consolidation courses (range, 6-30), corresponding to a median exposure time to bi-weekly nivolumab of 7.6 months (range, 2.3–15.5), displayed disease progression at the last follow-up. Conversely, in the study by Armand et al. and the ongoing trial of Baschier et al., a maximum of 8 consolidation courses with three-weekly pembrolizumab or 6 months of treatment with bi-weekly nivolumab, were respectively planned per protocol [25,28]. The optimal treatment duration of anti-PD1 monotherapy in patients with RR-HL who have failed ASCT remains controversial. While studies have suggested that a minor fraction, around 10–15%, of patients who achieve CR may maintain a long-term progression-free status upon continuous anti-PD1 monotherapy, most patients eventually progress after treatment discontinuation if not consolidated by allo-SCT [17,18,38,39]. Unfortunately, the search for robust predictive biomarkers to identify patients with a long-term response after anti-PD1 discontinuation has been mostly elusive [30,39,40,41]. Adopting anti-PD1 antibodies as a post-ASCT consolidation involves a biologically different situation due to the combined antitumor effects of HDT conditioning and transplant-related changes in PD1-expressing target immune cells [25,26]. Therefore, a fixed-duration therapy after ASCT may turn equally effective as a more prolonged treatment.

The management of patients with RR-HL is constantly evolving in the modern era [42]. The incorporation of novel agents, such as BV and PD1-blockers, in earlier treatment lines up to the frontline therapy, is generating significant changes in the strategic approach to patients with RR-HL [8,14,19,43]. While the survival outcomes of post-ASCT relapses are currently superior to those achieved over the last decade, there is still room for additional improvements [13,14,44]. In this scenario consolidation with PD-1 blockers may represent a further promising approach to counteract the risk of post-ASCT recurrence in specific subsets of patients.

## 5. Conclusions

While our study has some limitations related to its retrospective design, the relatively small size and heterogeneity of the study cohort, and the lack of centralized imaging assessments, we have provided the first real-life evidence supporting that post-transplant consolidation with PD-1 blockade is safe and associated with a conspicuous survival in patients at a very high-risk of ASCT failure. Further studies are warranted to define the most effective timing of PD1-blockade, i.e., as a bridge to ASCT or as a post-transplant consolidation, establish the optimal treatment length and better identify patients’ subsets more likely to benefit from this approach.

## Figures and Tables

**Table 1:** Summary of demographic and clinical characteristics of 26 patients with high-risk RR-HL who received post-ASCT consolidation with PD1-blockade.

Characteristics	N° (%)
	(N = 26)
*Age at transplant, (years)*	
Median (range)	30 (19–58)
*Gender*	
Male	15 (58)
Female	11 (42)
*Frontline therapy*	
ABVD	25 (96)
Stanford V	1 (4)
Consolidation radiotherapy	10 (38)
*Disease status after frontline therapy*	
Primary refractory	18 (69)
Relapse <12 months	4 (15)
Failure after interim PET-driven escalation (BEACOPP esc)	5 (19)
*Extranodal disease at relapse*	17 (65)
*B symptoms at relapse*	17 (65)
*Advanced stage at relapse (stage IVA-B, IIIA-B)*	17 (65)
*Number of salvage therapy lines*	
Median (range)	4 (1–5)
1 line	2 (8)
2 lines	5 (19)
>2 lines	19 (73)
*Exposure to BV pre-ASCT*	25 (96)
BV as single agent	21 (84)
BV and Bendamustine	4 (16)
Median number of BV cycles received (range)	4 (2–8)
*Exposure to anti-PD1 pre-ASCT*	16 (61)
Median number of anti-PD1 cycles received	11 (4–32)
*Exposure to BV and anti-PD1 pre-ASCT*	15 (58)
*Best response to last salvage therapy pre-ASCT*	
Not exposed to anti-PD1	10 (39)
CR	1 (10)
PR	7 (70)
SD/PD	2 (20)
Anti-PD1 as last salvage line before ASCT	16 (61)
CR	9 (56)
PR	7 (44)
SD/PD	---
*PET-CT status pre-ASCT*	
Negative (DS 1–3)	10 (38)
Positive (DS 4–5)	16 (62)
*Anti-PD1 post-ASCT* Median number of days from ASCT to anti-PD1 initiation Median number of anti-PD1 cycles received *Causes of anti-PD1 discontinuation (post-ASCT)*	44 (10–129) 13 (6–30)
CR (stop & follow-up)	18 (69)
Toxicity	0
Disease progression/recurrence/stability	5 (19)
Bridging to allo-SCT	1 (4)
No discontinuation	2 (8)

ABVD, doxorubicin, bleomycin, vinblastine, dacarbazine; BEACOPP esc, doxorubicin, cyclophosphamide, etoposide, procarbazine, prednisolone, bleomycin, vincristine; BV, brentuximab vedotin; ASCT, autologous stem cell transplantation; CR, complete remission; PR, partial remission; SD, stable disease; PD, progressive disease; allo-SCT, allogeneic stem cell transplantation.

## Data Availability

De-identified patient dataset is available from the corresponding author upon reasonable request.

## Supplementary Materials

The following supporting information can be downloaded at: https://www.mdpi.com/article/10.3390/cancers14235846/s1, Figure S1: Flowchart of the applied inclusion and exclusion criteria.
