# Supplementary figures and images for: Anti-PD1 Consolidation in Patients with Hodgkin Lymphoma at High Risk of Relapse after Autologous Stem Cell Transplantation: A Multicenter Real-Life Study
Source: Cancers (Basel). 2022 Nov 27;14(23):5846. doi: 10.3390/cancers14235846 (PMC9739754; doi:10.3390/cancers14235846)

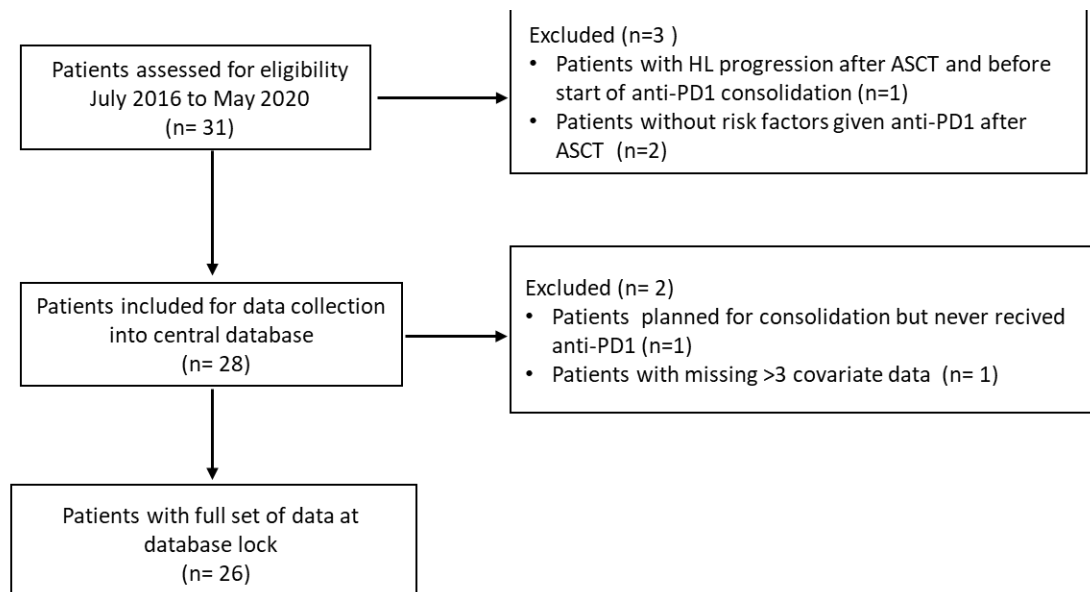

**Figure S1.** Flowchart of the applied inclusion and exclusion criteria.

Supplement: Supplementary file 1 [file cancers-14-05846-s001.zip › cancers-1998436-supplementary.pdf]
